# Borderline Personality Disorder Symptoms in College Students: The Complex Interplay between Alexithymia, Emotional Dysregulation and Rumination

**DOI:** 10.1371/journal.pone.0157294

**Published:** 2016-06-27

**Authors:** Rebecca Meaney, Penelope Hasking, Andrea Reupert

**Affiliations:** 1 Faculty of Education, Monash University, Melbourne, Victoria, Australia; 2 School of Psychology & Speech Pathology, Curtin University, Perth, Western Australia, Australia; 3 Department of Psychiatry, Monash University, Clayton, Victoria, Australia; University of Groningen, NETHERLANDS

## Abstract

Both Emotional Cascade Theory and Linehan’s Biosocial Theory suggest dysregulated behaviors associated with Borderline Personality Disorder (BPD) emerge, in part, because of cycles of rumination, poor emotional recognition and poor emotion regulation. In this study we examined relationships between rumination, alexithymia, and emotion regulation in predicting dysregulated behaviors associated with BPD (e.g. self-harm, substance use, aggression), and explored both indirect and moderating effects among these variables. The sample comprised 2261 college students who completed self-report measures of the aforementioned constructs. BPD symptoms, stress, family psychological illness, and alexithymia exerted direct effects on behaviors. Symptoms had an indirect effect on behaviors through rumination, alexithymia and emotional dysregulation. In addition, the relationship between symptoms and dysregulated behaviors was conditional on level of rumination and alexithymia. Implications for early identification and treatment of BPD and related behaviors in college settings are discussed.

## Introduction

Borderline Personality Disorder (BPD) impacts upon a wide range of cognitive and behavioral domains, resulting in symptoms such as intense dysphoric affect, chronic instability of mood, problematic interpersonal relationships, disturbed cognition and recurrent self-harm [1]. Rates of attempted suicide range between 38% to 73% in people with BPD, and 10% die by suicide, giving the disorder one of the highest mortality rates of all psychological conditions [2–4]. Of note, symptom severity peaks between the ages of 20 and 29 years, making this age group a particularly relevant target for intervention [5–8].

College students, the majority of whom are in this high-risk age group, report greater psychological distress, and psychiatric symptoms including symptoms of BPD, than non-students [9–12]. Although rates vary widely, up to 17.1% of college students have reported clinically significant symptoms of BPD [13]. In turn, despite having limited resources, college counselling services are increasingly being called upon to provide treatment for students with BPD [14–15]. Early and effective intervention demands a thorough understanding of how underlying risk factors work together to increase both symptom severity and behavioral markers of the disorder [13, 16].

Emotional Cascade Theory posits that aversive emotional states or symptoms induce rumination, which in turn increases the intensity of emotional distress, until dysregulated behaviors are employed as a mechanism of down-regulating, or reducing, distress [17]. This theory has previously been applied to explain dysregulated behaviors associated with BPD including self-injury, alcohol use and bulimic behaviors [18–20]. Consistent with Emotional Cascade Theory, rumination interacts with affective instability to predict self-injury [18], moderates the relationship between psychological distress and self-injury [21], interacts with BPD symptoms to predict dysregulated behaviors [22], and is related to BPD symptom severity [23–26].

Linehan’s [27] Biosocial Theory of BPD emphasizes the importance of emotion recognition and regulation in the development and maintenance of BPD. She adopts a broad view of emotion regulation, incorporating biological, cognitive and affective components that work together to effectively regulate emotional states. Further, Linehan argues that emotion regulation develops within the family context, with poor emotion regulation resulting, in part, from early invalidating environments [27]. Commensurate with this, a family history of psychological illness exacerbates risk for BPD, both through contribution to the BPD endophenotype, and the effect on family functioning [16]. Of note, caregivers with mental illness may be both less responsive to the emotional needs of their child, and less able to model adaptive emotional behaviors [28]. This may result in subsequent development of poor emotion recognition (alexithymia) [29] and impaired emotion management (emotional dysregulation) [30].

Alexithymia is characterized by diminished capacity to both identify and describe emotions, and consequently appropriately manage problematic emotional states [31–32]. As such, alexithymia is an underlying mechanism of emotional dysregulation [25,33]. Similarly, poor emotion regulation is noted among people with BPD [25–26]. Specifically, compared to those without the disorder, people with BPD employ significantly more expressive suppression (a response-focused strategy involving inhibiting the expression of distressing emotions) [34], and less cognitive reappraisal (an antecedent-focused strategy, whereby a potentially distressing event is interpreted in a manner that changes the impact of the emotions) [26, 35].

Much of the previous research concerning college students with BPD concentrates on exploring symptom severity [36]. However, while symptom severity is clearly an important indicator of the impact of the disorder, within a college environment minimizing the behaviors commonly engaged by people with BPD (e.g. substance abuse, self-injury, and physical attacks on others) also has implications for the safety of the broader college community [37]. In line with both Emotional Cascade Theory [17] and Linehan’s [27] Biosocial Theory of BPD, the presence of BPD symptoms induces regulatory cognitive strategies, thus employment of ineffective cognitive strategies (e.g. rumination and emotional dysregulation), may exacerbate symptom severity, and subsequently result in the use dysregulated behaviors as coping mechanisms [17, 27].

With reference to both Emotional Cascade Theory [17] and Linehan’s [27] Biosocial Theory of BPD, we aimed to clarify the roles of rumination, alexithymia, and emotional dysregulation in predicting dysregulated behaviors associated with BPD symptoms in college students. Specifically, we aimed to examine direct effects of family history of psychological illness, psychological distress, rumination, alexithymia and emotional dysregulation, on dysregulated behaviors, and indirect effects of the cognitive constructs on the relationship between symptoms and behaviors. Also in line with the aforementioned theories, we expected greater levels of rumination, alexithymia and poor emotion regulation would strengthen the relationship between BPD symptoms and behaviors.

## Materials and Methods

### Participants

A sample of 2261 college students was recruited from 28 Australian universities, across 6 Australian states and territories. The sample included 1642 women, 616 men, and 3 gender neutral (identify as neither male nor female) participants, who were between 18 and 77 years old (*M* = 24.82, *SD* = 8.05). The majority of participants were born in Australia (65.9%), and 8% stated they were of Aboriginal or Torres Strait Islander heritage. Overall, 74.8% were undergraduate students, and 84% had a full-time study load. Of the sample, 33.8% indicated a family history of psychological illness, with the most prevalent diagnosis being unipolar depression (57%). A total of 24.2% indicated a personal history of psychological illness, predominantly unipolar depression (61.2%). In the current sample, women and people of Aboriginal or Torres Strait Islander decent were over-represented relative to the national distribution of college students [38].

### Measures

The Borderline Symptom List (BSL-23) [39] is a self-report measure assessing symptoms of BPD, based on DSM-IV [40] criteria and the Diagnostic Interview for BPD—revised version [41]. The measure is unidimensional, and consists of 23 items that ask participants to rate how much they have experienced each symptom of BPD over the previous week, on a 5-point Likert scale ranging from 0 (not at all) to 4 (very much). Several researchers indicate a mean score of two or more on the BSL-23 is indicative of a level of symptom severity indicative of diagnosis of BPD, and a mean between 1.5 to < 2.0 representing sub-clinical symptoms of BPD [42–44]. The BSL-23 is reported by the authors as having good test-retest reliability over a one week period, (*r* = .82; *p* < .0001) [45], and high internal consistency (Cronbach’s alpha =. 93 - .97) [40]. In the current sample Cronbach’s alpha was .96.

The BSL-Supplement: Items for Assessing Behavior [39] is a 10-item self-report scale that assesses the frequency of specific behaviors over the previous week. Specifically, the supplement examines self-harming behaviors, suicidal intent and attempts, binge and purge behaviors, impulsivity, substance use, hostile outbursts, and sexual promiscuity. The behaviors on the supplement do not overlap with symptoms assessed with the BSL-23. The items are rated on a five-point frequency scale, with 0 (not at all) to 4 (daily or more often), and analyzed as mean scores. As expected, internal consistency of the behavior checklist in the current sample was moderate (α = .61) indicating potential differences in the types of behaviors engaged in.

The Ruminative Thought Style Questionnaire (RTS) [45] is a 20-item self-report scale that assesses for the presence of a ruminative thought style independent of the presence of depression. The RTS comprises a series of statements and the participant is asked to rate, on a 7-point Likert scale, how well the item describes them (0 = not at all; 7 = very well). The RTS consists of a single dimension, and the authors have reported high 2-week test-retest reliability (*r* = .80, *p* < .01) [46], and a Cronbach’s alpha of .95 (α = .92 in the current study).

The Toronto Alexithymia Scale (TAS-20) [47] is a self-report instrument containing 20 items assessing three core facets of alexithymia. The first factor, difficulty identifying feelings, consists of seven items that assess variations in ability to identify feelings, and distinguish them from somatic sensations associated with arousal. The second factor, difficulty describing feelings, contains five items examining the ability to describe feelings to other people. The remaining factor, externally-oriented thinking, contains eight items, and refers to a concrete, non-introspective cognitive style, or more simply a tendency to focus on external events over inner experiences.^48^ The format of the scale is a 5-point Likert with responses ranging from 1 (strongly disagree) to 5 (strongly agree). The authors report good test-retest reliability (.77, p < .01) and internal consistency (α = .81) [47], which was similar in the current sample (α = .77).

The Emotion Regulation Questionnaire (ERQ) [34] is a 10-item self-report scale that measures two strategies of emotional regulation: cognitive reappraisal and expressive suppression. The scale has six items on the appraisal factor, and four on the suppression factor, and is measured on a 7-point Likert scale with responses ranging from 1 (strongly disagree) to 7 (strongly agree). The scale is psychometrically sound, with Cronbach’s alpha ranging from .77 to .82 for the reappraisal factor, as was the case for the current sample (α = .81), and .68 to .76 for the suppression factor (α = .77 in current sample). Test-retest reliability across three months was .69 for both scales [34].

The Depression, Anxiety and Stress Scale (DASS) [48] is a 21-item self-report measure of symptoms of depression, anxiety and stress, which were statistically controlled for in the current study. The items ask participants to rate the degree to which they have experienced a specific characteristic of the three emotional states over the past week on a 4-point Likert scale ranging from 0 (did not apply to me at all) to 4 (applied to me very much or most of the time). Henry and Crawford [49] reported the DASS-21 to have high reliability overall (.93, 95% CI = .93 - .94), and across each of the subscales, Depression (.88, 95% CI = .87 - .89); Anxiety (.90, 95% CI = .89 - .91) and Stress (.93, 95% CI = .93 - .94) [49]. The current sample demonstrated similar reliability overall (α = .94), and for the Depression (α = .88), Anxiety (α = .90), and Stress (α = .87), subscales.

Participants were also asked to state their current age, their gender, and whether any family member had been diagnosed with a history of psychological illness.

### Procedure

Ethical approval to conduct this project was obtained from the Monash University Human Research Ethics Committee.

Participants were recruited through fliers advertising the research, as well as a Facebook Community page that contained the link to the online questionnaire. Australian college webmasters were also asked to place messages on their college webpages directing interested students to the questionnaire. In all cases the advertisement explained the purpose of the research, scope, time involved and incentive, in addition to a link to the online questionnaire. All participants were informed of the voluntary nature of participation and confidentiality of data. Interested participants provided their email addresses to enter a draw to win an iPad valued at AU$500. Contact details were stored separately from questionnaire responses and deleted immediately after the prize draw. Consent to participate was implied by completing the questionnaire.

### Data analysis

We sought to explore the relationship between BPD symptoms and behaviors, and whether rumination, alexithymia, and/or emotion regulation mediated or moderated this effect. Assumptions related to the following analyses were met. Overall there was a sufficient ratio of cases to predictors [50], and no multivariate outliers were identified (*x*^*2*^ = 37.70 for *df* = 15, α = .001). Hayes’ [51] PROCESS Macro for SPSS was used to assess the magnitude and significance of the direct and indirect effects of the predictor variables on the criterion, with 5000 bootstrapped re-samples, and significance determined on 95% bias corrected confidence interval (CI). Continuous predictors were mean-centered prior to analysis; direct and indirect effects were assessed prior to examining moderated effects at ± 1SD from the mean [51].

We entered BPD behaviors as the outcome, with BSL-23 scores (BPD symptoms) as the predictor, and rumination, alexithymia, and emotional regulation as potential mediators and moderators of this relationship. Participant gender, age, psychological distress (depression, anxiety and stress), and family history of psychological illness were entered as covariates. As data was collected from multiple sites, geographic location was included as a potential covariate in the analyses; however this had no effect on the data and thus was excluded from the reported analyses. Where applicable, results that lost interpretative validity when rounded to two decimal places (e.g. .003) are reported at three decimal places. The data used in the following analyses may be viewed on the Figshare repository.

## Results

### Descriptive data and relationships between variables

Of the sample, 8.1% (n = 197) met the diagnostic cut-off for BPD (mean BSL-23 score > 2.0), predominantly represented by females (77%, n = 150). In turn, 22.5% (n = 509) of the sample reported clinically relevant sub-diagnostic symptoms (mean BSL-23 = 1.5 - < 2), also primarily females (72%, n = 367). No gender differences were observed on symptoms *t*(2165) = 1.94, *p =* .052, but females (*M* = 2.7, SD = 3.1) were more likely to report engaging in BPD behaviors than males (*M* = 2.2, SD = 3.0); *t*(2013) = 2.86, *p =* .020. Specifically, our results indicated that female students with BPD are more likely to report having behaved in an aggressive manner (46%), and engage in self-harm (38%) compared to males with the disorder (aggression 25%, self-harm 25%), and other students without BPD (aggression for females 12.2%, males 9.1%; self-harm for females 4.7% and males 4.9%). Females below the BPD cut-off primarily reported binge eating (43.9%), and getting drunk (43.6%). Males from both groups most frequently reported getting drunk (above cut-off: 52.2%; below cut-off: 46.9%), followed by binge eating (above cut-off: 41.3%; below cut-off: 33.4%). The frequencies of behaviors across all aforementioned groups are shown in Table 1, while sample descriptive statistics and correlations between variables are presented in Table 2.

**Table 1:** BPD behaviors engaged in by group and gender.

	Above BPD cut-off^1^		Below BPD cut-off^2^	
*Behavior over the past week*^3^	Females %	Males %	Females %	Males %
Angry outbursts/attacked others	46.0^4^	25.0	12.2	9.1
Engaged in self-harm	38.0	27.3	4.7	4.9
Got drunk	36.4	52.2	43.6	46.9
Problematic sexual encounters	32.4	18.5	16.2	7.1
Binge eating episodes	31.7	41.3	43.9	33.4
High-risk behaviors	31.2	29.3	13.2	15.1
Medication misuse/overdose	27.0	25.0	7.6	7.1
Purging after eating	26.1	6.8	7.7	1.9
Expressed suicidal intent to others	20.7	27.3	3.0	3.0
Used drugs	13.4	22.7	7.3	10.4
Attempted suicide	8.1	9.1	0.5	0.6

Note:

^1^ Participants (n = 197) with mean score of ≥ 2 on the Borderline Symptom List– 23 (BSL-23);

^2^ Participants (n = 2064) with mean score < 2 on BSL-23;

^3^ Behavior engaged in at least once over the previous week;

^4^ Most frequent behaviors shown in bold font.

**Table 2:** Correlations and descriptive statistics for key variables.

Variable	Mean	*SD*	2.	3.	4.	5.	6.	7.	8.	9.	10.	11.	12.	13.	14.
1. Age	24.82	8.05	.02	.08***	-.07**	-.07**	.01	-.08***	-.09***	-.16***	.10***	-.13***	-.10***	-.14***	.010
2. Gender	-	-	-	-.11***	.03	-.02	-.09***	-.03	-.07***	-.05*	-.05*	.17***	-.01	.09***	-.01
3. Family history	-	-		-	.15***	.08***	.18***	.18***	.19***	.14***	-.05*	-.02	.11***	.07**	-.01
4. Depression	12.40	10.44			-	.67***	.69***	.82***	.41***	.47***	-.31***	.28***	.49***	.33***	.02
5. Anxiety	9.76	8.57				-	.73***	.71***	.38***	.46***	-.23***	.21***	.50***	.31***	.09***
6. Stress	14.02	9.51					-	.70***	.43***	.51***	-.26***	.13***	.47***	.26***	.06*
7. BPD symptoms	19.35	16.59						-	.53***	.54***	-.30***	.29***	.58***	.37***	.05*
8. BPD behavior	2.57	3.11							-	.34***	-.19***	.10***	.38***	.22***	.04
9. Rumination	67.73	12.81								-	.29***	.55***	.55***	.44***	.14***
10. Cog. Reappr.^1^	28.38	6.10									-	-.05*	-.25***	-.15***	.18***
11. Expr.Supp.^2^	15.38	4.88										-	.40***	.57***	.10***
12. DIF^3^	17.31	5.97											-	.64***	.15***
13. DDF^4^	14.30	2.90												-	.20***
14. EOT^5^	25.40	2.97													-

*** Significant at < .001 level (2-tailed);

** Significant at .01 level (2-tailed);

* Significant at .05 level (2-tailed);

^1^ Cognitive reappraisal (emotional regulation);

^2^ Expressive Suppression (emotional regulation);

^3^ Difficulty identifying feelings (alexithymia);

^4^ Difficulty describing feelings (alexithymia);

^5^ Externally oriented thinking (alexithymia).

### Predicting BPD behaviors

Mediation analysis: BPD symptoms, having a family history of psychological illness, reporting higher levels of stress, and having difficulty identifying feelings all exerted direct effects on dysregulated behaviors (Table 3). BPD symptoms had an indirect effect on behaviors through engaging in rumination, *B* = .23, *SE* = .03, 95% CI: .18 - .29, κ^2^ = .02, difficulty identifying feelings, *B* = .16, *SE* = .01, 95% CI: .14 - .19, κ^2^ = .01), difficulty describing feelings, *B* = .04, *SE* = .01, 95% CI: .03 - .06, κ^2^ = .01, and expressive suppression, *B* = .07, *SE* = .01, 95% CI: .04 - .09, κ^2^ = .01. A calculation of the proportion of maximum possible indirect effect (κ^2^ index) indicated all results were associated with a small effect size (small = .01, medium = .09, large = .25) [52].

**Table 3:** Predictors of BPD behaviors and BPD symptoms.

Variable	B	SEB	95%CI Lower	95%CI Upper	R^2^	F
					.33***	47.53
BPD Symptoms	.08***	.008	.06	.09		
Age	-.02*	.08	-.04	-.01		
Gender	-.01	.14	-.37	-.01		
Family history	.50**	.13	.26	.75		
Depression	-.06*	.02	-.11	-.02		
Anxiety	-.04	.03	-.08	.01		
Stress	.08**	.02	.03	.12		
Difficulty identifying feelings	.05**	.02	.02	.08		
Difficulty describing feelings	.02	.03	-.04	.08		
Externally oriented thinking	.003	.02	-.04	.04		
Cognitive reappraisal	-.002	.01	-.02	.02		
Expressive suppression	-.05**	.02	-.08	-.02		
Rumination	.01	.01	-.000	.02		

*p< .05

**p < .01

***p< .001

**Fig 1:**
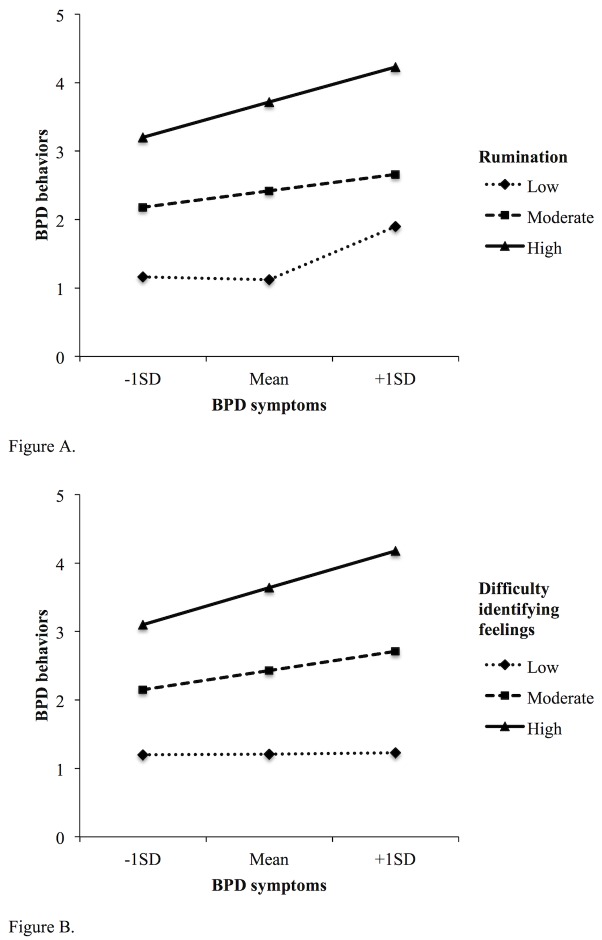
The increasing magnitude of effect BPD symptoms have on behaviors solely at moderate and high levels of rumination (A), and difficulty identifying feelings (B).

Moderation analysis: Significant conditional effects of symptoms on behaviors were found for rumination, *B* = .000, *SE* = .000, 95% CI: .000 - .001, and difficulty identifying feelings, *B* = .000, *SE* = .000, 95% CI: .000 - .001. As may be seen in Fig 1A, there was no relationship between symptoms and behaviors at low, *B* = *-*.001, *SE* = .002, 95% CI: -.004 - .002, or moderate levels of rumination, *B* = .003, *SE* = .002, 95% CI: -.002-.006, but a positive relationship was observed at high levels of rumination, *B* = .007, *SE* = .003, 95% CI: .000 - .013. As shown in Fig 1B, no relationship between symptoms and behaviors was observed a low levels of difficulty identifying feelings, *B* = .001, *SE* = .003, 95% CI: -.006 - .006, however positive relationships were found at both moderate, *B* = .008, *SE* = .002, 95% CI: .004 - .013, and high levels, *B* = .015, *SE* = .004, 95% CI: .007 - .024.

## Discussion

Emotional Cascade Theory [17] posits that interactions between emotional and behavioral dysregulation occurs through ‘emotional cascades’ whereby rumination increases emotional distress, and dysregulated behaviors are employed as a means of down-regulation. Similarly, Linehan [27] argues the importance of emotion recognition and regulation in the development of BPD and associated behaviors. In this study we examined relationships between rumination, alexithymia, and emotion regulation in predicting BPD behaviors, and explored both indirect and moderating effects among these variables. Consistent with the above theories, dysregulated behaviors associated with BPD were related to engaging in rumination, having difficulty identifying feelings and difficulty describing feelings, and engaging in expressive suppression. Specifically, difficulty describing feelings and suppression mediated the relationship between symptoms and behaviors, while rumination and difficulty identifying feelings both mediated and moderated the relationship. While confirming the salience of these variables in emotionally dysregulated behaviors, our findings also highlight the complexity in these relationships, and underscore the need for a more nuanced understanding of these behaviors in a college context.

The observed BPD rate of 8.1% is higher than previously reported in age matched general population samples (e.g. 4–6%) [5, 53]. Given nearly a quarter of our sample endorsed having a history of psychological illness, sample bias is a possibility, however the finding may also lend support to the assertion that college students experience higher levels of psychological distress, and symptoms of BPD, than their non-studying counterparts [9–10, 12]. Our rate of BPD aligns with numerous other studies of college students, both in terms of diagnostically relevant BPD (e.g. 9.9%; 7.9%; 8.5%) [54–56], and subclinical symptoms (e.g. 18.6%; 25.5%) [35, 57]. In turn, we found that nearly half of female students with BPD engaged in aggressive behavior, and over a third in self-harm, while over half of the male students with BPD reported getting drunk. While we did not ask participants the location of these behaviors (i.e. college or elsewhere), should even a small number of these events occur on campus, other students and staff may be at risk of aversive outcomes due to exposure to student aggression or intoxication. Together, our results suggest behaviors typical of BPD are highly prevalent among college students, and should they occur on campus, have the potential to impart significant burden on college staff, and potentially other students.

The observed relationships between BPD symptoms, behaviors, and the constructs we examined are generally consistent with previous work [1, 17, 20, 22, 24, 27, 58]. The salience of rumination and alexithymia align with Selby and Linehan’s theories [17, 22, 27], yet our findings suggest severity of both BPD symptoms and cognitive factors also play an important role. Specifically, symptom severity appears related to the severity of rumination, alexithymia and emotional suppression, which in turn is related to frequency of dysregulated behavior. Further, lower levels of alexithymia and rumination appear to have little effect on dysregulated behaviors, but the relationship between symptoms and behaviors is rapidly exacerbated as both alexithymia and rumination increase. While these results were associated with a small effect size, Preacher and Kelley [52] purport that mediator variables generally yield a small effect size. As such, Preacher and Kelley recommend interpretation of any effects in terms of substantive importance rather than arbitrary statistical benchmarks.

We consider our findings may have utility in conceptualizing the cognitive mechanisms that predict dysregulated behaviors, and possibly, another key characteristic of the disorder. Poor distress tolerance is an endophenotype of BPD [27], and our findings may contribute to understanding the factors that maintain this feature of BPD. Under the auspice of Biosocial [27] and Emotional Cascades Theory [17], rumination, alexithymia, and emotional suppression are considered as cognitive dysregulation, employed in response to the presence of BPD symptoms. Our results suggest that higher levels of rumination, and difficulty identifying feelings, ultimately amplify the prominence of symptoms. Similarly, emotional suppression requires considerable cognitive effort, yet is ineffective in managing distress [25, 33]. As such, the presence of BPD symptoms may result in employment of ineffective down-regulating strategies, which in turn may result in the person with BPD perceiving poor self-efficacy in managing, thus tolerating, emotional distress.

### Implications

Further research is required to clarify the exact role of rumination, alexithymia and emotion regulation in initiating and maintaining both BPD symptoms and dysregulated behaviors. Nonetheless, the present findings underscore the predictive power of rumination, alexithymia and emotional dysregulation in exacerbating the relationship between BPD symptoms and behaviors. This finding suggests college-based treatment programs for students with BPD could utilize components of existing therapies. For example, skills specific to Dialectical Behavior Therapy, such as distress tolerance, distraction techniques, and improving emotional awareness [27], have already shown promising results within a college mental health setting [14].

College services may benefit from confirmation that dysregulated behaviors associated with BPD represent an identifiable challenge in student populations. While the behaviors we examined are not unique to BPD, we found that students with BPD have a greater likelihood of engagement. This finding suggests the utility of both college-based prevention initiatives to assist students manage these behaviors, and guidelines for college staff to manage related behavioral incidents effectively. In turn, it may be the case that alexithymia, rumination and emotional suppression influence engagement in a specific dysregulated behavior. Future research could attempt to examine more specific effects.

### Limitations

There were several limitations to this study. Despite having good psychometric properties the measure of BPD encompassed a number of symptoms, some common to other psychological disorders such as depression and anxiety. Subsequently, while we controlled for depression, anxiety and stress, we cannot assert that the relationships we observed are unique to BPD. Similarly, rumination was measured as a general construct, however content-specific rumination (e.g., depressive rumination) may bear a differential influence in both symptoms and behaviors associated with BPD. Other measures of emotion regulation such as the Difficulties in Emotion Regulation Scale (DERS) [59], tap a wider range of constructs such as emotional awareness, acceptance of emotions, presence of goal directed behavior, and access to effective regulatory strategies and could fruitfully be used in future.

Both the cross-sectional design and bias associated with self-report measures suggest caution is required when interpreting the clinical validity of BPD symptoms and behaviors. Longitudinal research is also needed to delineate the temporal associations between the constructs. Such work would provide key insights into salient targets for early intervention to reduce symptom severity and reduce BPD-related behaviors on campus. Of importance, the study does not distinguish the protective factors that serve to differentiate students with BPD that are functioning academically and socially despite the presence of symptoms. Further investigation of protective factors bears particular importance, as it may be the case that these factors can be incorporated into treatment programs.

## Conclusions

Findings of the current study are important given they suggest that symptoms of BPD are apparent in college students, and associated with higher levels of psychological distress and high-risk behaviors. This suggests the need for colleges to allocate resources for prevention, early intervention, and subsequent treatment. In turn, we consider that our findings add to the depth of understanding toward Emotional Cascade Theory, and to a lesser degree, Biosocial Theory, through demonstrating that rumination, alexithymia and emotional dysregulation differ in the degree of influence toward the relationship between behaviors and symptoms. As college counseling services may increasingly be required to provide clinical interventions for students with BPD, we hope that our findings contribute to the confidence of such services in their ability to service this student population.
